# The moderating effect of psychological capital on the relationship between nurses’ perceived workplace bullying and emotional exhaustion: a cross-sectional study

**DOI:** 10.1186/s12912-025-02763-0

**Published:** 2025-01-28

**Authors:** Xiao Peng, Jing Ma, Ying Chen, Ying Han, Hong Zhou, Aiping Gong, Fang Peng, Xinzhang Sun, Xingfen Wang, Xunya Xiong, Li Li, Mengting Huang, Qingsong Zeng

**Affiliations:** 1 Department of Obstetrics and Gynecology, The First Affiliated Hospital of Yangtze University, Jingzhou, Hubei Province China; 2 Yangtze University Health Science Center, Jingzhou, Hubei Province China; 3 Pingshan District People’s Hospital of Shenzhen, Shenzhen, China; 4 Department of Gastroenterology, Xianning City Central Hospital, Xianning, Hubei Province China; 5 Department of Gynecology, The First Affiliated Hospital of Yangtze University, Jingzhou, Hubei Province China; 6 Oncology Department, Xianning City Central Hospital, Xianning, Hubei Province China; 7 Oncology Department, The First Affiliated Hospital of Yangtze University, Jingzhou, Hubei Province China; 8 School of Nursing and Midwifery, Monash University, Clayton, VIC Australia

**Keywords:** Emotional exhaustion, Workplace bullying, Psychological capital, Nurses, Moderating effect

## Abstract

**Background:**

Workplace bullying (WPB) is common in nursing profession, leading to adverse effects on nurses’ health and teamwork. Although it has been suggested that psychological capital (PsyCap) could potentially moderate the relationship between WPB and emotional exhaustion, there is currently a lack of direct empirical evidence supporting this claim. Therefore, this study aims to examine how PsyCap moderates the relationship between WPB and emotional exhaustion in nurses.

**Methods:**

A cross-sectional study was carried out involving 1068 nurses using a general information questionnaire, Negative Acts Questionnaire-Revised, Psychological Capital Questionnaire-Revision, and emotional exhaustion subscale of the Chinese version of Maslach Burnout Inventory-General Survey. The PROCESS macro was utilized to examine the moderating effect of PsyCap.

**Results:**

WPB led to emotional exhaustion among nurses (β = 1.488, *P* < 0.001), and PsyCap moderated this positive relationship (β = 0.300, *P* < 0.001). The group with high PsyCap exhibited lower levels of emotional exhaustion. However, as the perceived WPB increased, the disparity in emotional exhaustion between the high and low PsyCap groups diminished.

**Conclusions:**

WPB significantly contributes to nurses’ emotional exhaustion. PsyCap mitigates this impact, but this effect is limited in organizations with high WPB. it is recommended that nursing managers mitigate the detrimental impact of WPB on nurses’ emotional well-being by both strengthening nurses’ individual PsyCap and implementing comprehensive strategies to reduce WPB behaviors.

## Introduction

Workplace bullying (WPB) is widely acknowledged as a pervasive issue faced by the nursing profession [1]. It involves ongoing and recurring negative behaviors, whether real or perceived, executed by individuals or groups in positions of authority. Victims of WPB often face challenges in effectively protecting themselves over prolonged periods [2]. Bullying behaviors include but are not limited to unwarranted criticism, verbal assaults, refusal to help, persistent undermining of achievements, disregard for opinions, provocation, intimidation and the utilization of threats or biased penalties [3]. A systematic review found the global prevalence of WPB among nurses to be approximately 30.8% [4]. In a study conducted by Peng et al. [5], which surveyed over 5000 Chinese nurses, it was reported that 30.75% had encountered WPB within the preceding six months.

Previous research has shown that WPB negatively impacts the psychological and physical well-being of bullied nurses [6]. It also hinders effective teamwork and communication among nursing professionals and other team members, which ultimately threatens the quality of nursing care and compromises patient safety [6]. It is noteworthy that emotional exhaustion often emerges as the initial negative response experienced by nurses subjected to WPB, thereby triggering the aforementioned negative consequences. Emotional exhaustion is defined as the feeling of being emotionally overextended and drained by one’s work, which is regarded as the core element of job burnout [7, 8]. According to the study of Ma et al., it is suggested that emotional exhaustion may serve as a mediator in the relationship between WPB and nurses’ affective occupational commitment [9]. Likewise, Srivastava et al. [10] revealed that emotional exhaustion plays a mediating role between WPB and workers’ intention to leave. Therefore, it can be inferred that WPB contributes to emotional exhaustion among nurses.

The theory of Job Demand-Resource (JD-R) provides a heuristic way for viewing how job characteristics influence the employees’ emotional exhaustion. As per the JD-R theory, when employees face high job demands and limited job resources, their chances of experiencing emotional exhaustion are heightened [11]. Previous studies have demonstrated that WPB can be considered as a type of job demand within the JD-R framework, which contributes to employees’ emotional exhaustion and depletes the available resources that could be better used to achieve work goals [12]. Additionally, the affective event theory (AET) posits that an employee’s experience of positive or negative work events elicits individual affective responses (which refer to employees’ moods and emotions), which in turn influence their attitudes and behaviors towards work. WPB can be regarded as a negative work event or stressful situation that may cause negative affective responses, such as emotional exhaustion [13]. Numerous studies have consistently demonstrated a positive correlation between WPB and emotional exhaustion [14–17]. Given the high prevalence of WPB in nursing profession and its serious consequences, it is imperative to explore protective factors that can help reduce the impact of WPB on nurses’ emotional exhaustion.

Psychological capital (PsyCap) is a noteworthy factor that refers to an individual’s optimistic psychological state of development, encompassing positive psychological constructs such as hope, efficacy, resilience, and optimism [18]. PsyCap has been recognized as a crucial resource that empowers individuals to effectively cope with stressors and adversities in the workplace [19]. Introducing PsyCap into the framework of WPB and its consequences, particularly emotional exhaustion, offers a promising avenue for understanding how nurses might mitigate the negative impact of bullying experiences. According to AET, personal traits moderate the effect of work events (such as WPB) on affective responses (like emotional exhaustion) [13]. Since PsyCap falls within the realm of personal traits, it implies that PsyCap could potentially moderate the relationship between WPB and emotional exhaustion. The JD-R theory also suggests that job resources have the potential to shield employees from the adverse effects of excessive job demands, implying that PsyCap might mitigate emotional exhaustion caused by WPB. However, there is currently a lack of direct empirical evidence supporting this claim. By clarifying the role of PsyCap in the relationship between nurses’ perception of WPB and emotional exhaustion, we can gain a deeper understanding of the boundary effects within this relationship. Furthermore, this insight is critical for developing strategies aimed at mitigating the negative impacts of WPB and effectively managing emotional exhaustion among nurses.

**Fig. 1:**
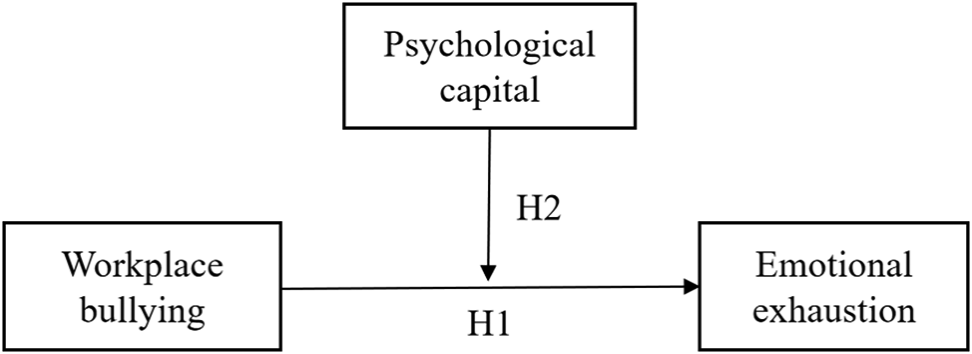
Theoretical hypothesis model

Therefore, the objective of this study was to examine how nurses’ perceived WPB affects their emotional exhaustion and whether PsyCap has a moderating effect on this association. Based on the aforementioned evidence, we postulate that: (1) nurses’ perceived WPB has a positive impact on their emotional exhaustion; and (2) PsyCap has a moderating effect on the relationship between WPB and emotional exhaustion. The hypothetical model we developed is shown in Fig. 1.

## Methods

### Participants and procedure

A cross-sectional descriptive study was carried out in two general tertiary hospitals (> 500 beds) situated in the cities of Jingzhou and Xianning in the Chinese province of Hubei. A convenience sampling method was used to recruit eligible registered nurses from June and July 2022. To ensure the representativeness of the sample, we included nurses from various departments, such as internal unit, surgical unit, obstetrics and gynecology, pediatrics, emergency room, and intensive care unit (ICU) in our sampling process. The participants were required to meet the following criteria: (a) be a registered nurse employed at a hospital; (b) have worked for at least 6 months; and (c) agree to take part in the survey anonymously. Nurse managers were excluded from the sample. The minimum required sample size was calculated using the formula for cross-sectional designs [20]:$\:\:n={[z}_{1-\alpha\:/2}^{2}\times\:p\times\:(1-p)]/{\delta\:}^{2}$, with *α* representing the type I error (*α* = 0.05), *z*_1-*a*/2_ as the confidence level (*z*_1-*a*/2_=1.96), *p* as the parameter in sample calculation, and *δ* as the margin of error (*δ* = 0.03 in this study). According to the previous study, the prevalence of WPB among Chinese nurses was 46.20% [21]. Therefore, in this study, *p* was set at 46.20%. Based on the provided formula, it is expected that the study will require a sample size of 1061.

Data were gathered through the utilization of Wenjuanxing, a popular online survey tool in China available at https://www.wjx.cn/. Prior to their participation, all participants were provided with detailed information about the purpose, benefits, risks, anonymity, voluntary nature of participation, and their right to withdraw. Informed consent forms and survey links were distributed through popular instant messaging applications in China such as WeChat, Ding Talk or Short Message Service. Nurses accessed the survey by clicking on the link or scanning the QR code provided. The researchers also clarified that completing and returning the questionnaires would indicate their consent to participate. To prevent duplication, each IP address was limited to submitting only one questionnaire response. Ethical approval was provided by the Ethics Committee of Health Science Center, Yangtze University (No. 202102015). Out of the 1085 registered nurses who agreed to take part in the survey, all completed the questionnaires. However, 17 questionnaires were excluded during data double-checking due to an obvious regular response. Thus, a total of 1068 valid questionnaires were collected in the study, resulting in a valid response rate of 98.40% (1068/1085).

### Measures

#### Demographic information

Demographic data such as age, gender, marital status, educational level, years of experience in nursing, professional title, contract status, type of unit, personal monthly income, and number of night shifts per month were gathered from all participants.

#### Perceived WPB

In this study, the Negative Acts Questionnaire-Revised (NAQ-R) in its Chinese version was utilized. The scale comprises 22 items and has been translated and validated by Xun et al., based on Enarsen et al.’s English version [22, 23]. It encompasses three dimensions: person-related negative acts, work-related negative acts and organizational injustice. Participants were asked to complete a self-report survey employing a 5-point Likert scale, ranging from “1 = never” to “5 = almost every day”. They were instructed to evaluate the frequency at which they perceived specific negative behaviors exhibited by their nursing colleagues within the previous six-month period. The overall scores varied between 22 and 110, with scores below 32 indicating “not bullied”, scores between 33 and 44 suggesting “occasional bullied”, and scores above 45 indicating “severely bullied” [24]. The validation of the scale has been effectively demonstrated among Chinese nurses [22]. The tool exhibited a Cronbach’s α coefficient of 0.950 in this study.

#### PsyCap

The Chinese version of the Psychological Capital Questionnaire-Revision (PCQ-R), comprising 20 items, was employed in this study[18^,^ 25]. This instrument comprises four dimensions: efficacy, hope, resilience, and optimism. Every individual item was assessed with a 6-point Likert scale, with responses ranging from “1 = strongly disagree” to “6 = strongly agree”, leading to a cumulative score between 20 and 120. A higher overall score signifies a higher level of PsyCap. The tool showed an adequate reliability and validity in the nursing population [25]. The Cronbach’s α coefficient of this scale was 0.984 in this study.

#### Emotional exhaustion

Nurses’ level of emotional exhaustion was evaluated using the emotional exhaustion subscale of the Chinese version of the Maslach Burnout Inventory-General Survey (MBI­-GS) [26]. There were 5 items in the subscale, with each item being scored on a scale from 0 (never) to 6 (very frequently). The range of the overall score was 0 to 30, with higher scores indicating higher levels of emotional exhaustion. This subscale has previously demonstrated excellent internal consistency among Chinese nurses [27]. The study revealed a Cronbach’s α coefficient of 0.954 for the subscale.

### Data analysis

SPSS 26.0 software was utilized for data analysis. Descriptive statistics such as mean, standard deviations, frequency or percentages were utilized to describe the demographic information and scores of the study variables. To examine the differences in emotional exhaustion based on demographic characteristics, independent t-tests and analysis of variance (One-Way ANOVA) were employed. Pearson correlation tests were performed to examine the relationships between nurses’ perceived WPB, PsyCap, and emotional exhaustion.

The moderation analysis was performed by employing the SPSS PROCESS V3.5 macro developed by Hayes. Model 1 was utilized to test the moderating role of PsyCap in relation to the impact of nurse’ perceived WPB on their emotional exhaustion [28]. The pick-a-point method was conducted to test the significance of the moderation effect. The high PsyCap group (M + 1SD) and low PsyCap group (M-1SD) were defined based on the moderating variable of PsyCap, with one standard deviation above or below the mean. The covariates that showed significant associations with emotional exhaustion in the univariate analyses were taken into account for the moderation model analyses. All variables were centered prior to inclusion in the model. The reported *P* values were two-tailed, and statistical significance was defined as *P* < 0.05.

### Common method bias

To evaluate the presence of common method bias (CMB) in the collected data, a Harman’s single-factor test was conducted using factor analysis [29]. The analysis indicated that the first factor accounted for only 41.42% of the total variance, which is below the recommended threshold of 50% [29, 30]. This finding suggests that CMB does not substantially affect the validity of the results in this study.

## Results

### Demographic characteristics and univariate analyses

Participants’ age varied from 20 to 60 years old (Mean = 31.69, SD = 6.23), with nursing experience ranging from 1 to 36 years (Mean = 9.89, SD = 6.78). Other detailed demographic information is shown in Table 1. Univariate analyses showed significant differences in the emotional exhaustion score for demographic characteristics of age, type of unit, night shifts per month (*P* < 0.001) (Table 1).

**Table 1:** Demographic characteristics of participants and univariate analyses between these characteristics and emotional exhaustion (*n* = 1068)

Variable	*n* (%)	Emotional Exhaustion (M ± SD)	t / F value	*P*
Age (years)			6.119^2)^	0.002
20-	440 (41.2)	9.17 ± 6.45		
30-	504 (47.2)	9.09 ± 5.63		
40-	124 (11.6)	7.15 ± 4.96		
Gender			-1.228^1)^	0.224
Female	1005 (94.1)	8.82 ± 5.78		
Male	63 (5.9)	10.08 ± 8.00		
Marital status			2.768^2)^	0.063
Unmarried	290 (27.2)	9.52 ± 6.58		
Married	756 (70.8)	8.70 ± 5.69		
Windowed and divorced	22 (2.1)	7.32 ± 4.81		
Education			-1.032^1)^	0.302
Secondary or advanced diploma	68 (6.4)	8.18 ± 6.44		
Bachelor’s degree or above	1000 (93.6)	8.95 ± 5.90		
Years of experience			2.192^2)^	0.112
< 6	298 (27.9)	9.10 ± 6.52		
6-	378 (35.4)	9.25 ± 6.09		
11-	392 (36.7)	8.40 ± 5.94		
Professional title			2.289^2)^	0.102
Nurse	153 (14.3)	8.56 ± 6.74		
Nurse practitioner	499 (46.7)	9.31 ± 6.16		
Nurse-in-charge and above	416 (39.0)	8.52 ± 5.30		
Contract status			-1.535^1)^	0.125
Permanent	369 (34.6)	8.53 ± 5.20		
Temporary	699 (65.4)	9.09 ± 6.29		
Type of unit			4.539^2)^	< 0.001
Medicine unit	376 (35.2)	9.76 ± 6.52		
Surgical unit	232 (21.7)	9.19 ± 6.18		
Obstetrics/ gynaecology	91 (8.5)	7.71 ± 5.33		
Paediatrics	97 (9.1)	7.27 ± 4.84		
Emergency room/ ICU	96 (9.0)	8.97 ± 5.22		
Other	176 (16.5)	8.13 ± 5.19		
Personal monthly income (¥)			0.465^2)^	0.628
< 6,000	342 (32.0)	8.65 ± 6.83		
6,000-	703 (65.8)	9.01 ± 5.47		
10,000-	23 (2.2)	9.26 ± 5.80		
Number of night shifts per month			3.365^2)^	0.010
0	105 (9.8)	8.03 ± 4.86		
1–4	307 (28.7)	8.41 ± 5.95		
5–9	442 (41.4)	9.21 ± 6.07		
≥ 10	147 (13.8)	10.07 ± 6.21		
During pregnancy or lactation	67 (6.3)	7.84 ± 5.50		

Abbreviations: ICU, Intensive care unit. ^1)^ t value; ^2)^ F value

### Descriptive statistics and correlations among the study variables

The mean total scores for WPB, PsyCap and emotional exhaustion were 28.87 ± 8.92, 91.59 ± 19.20, 8.90 ± 5.94, respectively; the mean item scores for WPB, PsyCap and emotional exhaustion were 1.31 ± 0.41, 4.58 ± 0.96, 1.78 ± 1.19, respectively (Table 2). For perceived WPB, a total of 812 (76.0%) nurses scored below 33, while 256 (24.0%) nurses scored 33 or above, showing a 24.0% prevalence rate of WPB among nurses over the previous six months. This comprised of 199 (18.6%) of nurses who experienced “occasionally bulliedd” and 57 (5.4%) who encountered “severely bullied”. From the Pearson correlation analysis, there was a positive association between nurses’ perceived WPB and emotional exhaustion (*r* = 0.542, *P* < 0.01); Additionally, PsyCap showed negative correlations with both WPB and emotional exhaustion (*r* = -0.316, *P* < 0.01; *r* = -0.393, *P* < 0.01).

**Table 2:** Descriptive statistics and correlations among study variables

Variable	Total score			Item score		1	2	3
	M ± SD	Range		M ± SD	Range			
1.WPB	28.87 ± 8.92	22–94		1.31 ± 0.41	1-4.27	1		
2.PsyCap	91.59 ± 19.20	20–120		4.58 ± 0.96	1–6	-0.316^**^	1	
3.Emotional exhaustion	8.90 ± 5.94	0–30		1.78 ± 1.19	0–6	0.542^**^	-0.393^**^	1

*Note*: M, mean; SD, standard deviation; WPB, workplace bullying; PsyCap, psychological capital; ^**^P < 0.01

### Impact of WPB on emotional exhaustion and the moderating effect of PsyCap

With regard to hypothesis 1, after controlling for age, type of unit, and night shifts per month, a significant positive association was found between WPB and emotional exhaustion (*β* = 1.488, *P* < 0.001) (Table 3). Regarding hypothesis 2, the relationship between WPB and emotional exhaustion was moderated by PsyCap (interactive effect = 0.300, *P* < 0.001). The pick-a-point method showed that the impact of nurses’ perceived WPB on emotional exhaustion was statistically significant at low (*β* = 1.200, SE = 0.083, 95%CI 1.037, 1.364), moderate (*β* = 1.488, SE = 0.079, 95%CI 1.332, 1.643), and high levels (*β* = 1.775, SE = 0.114, 95%CI 1.551, 2.000) of PsyCap, further corroborating the significant moderating effect of PsyCap in the relationship between WPB and emotional exhaustion (Table 4).

**Table 3:** Impact of WPB on emotional exhaustion and moderating effect of PsyCap

Variables	Emotional exhaustion					
	β	Boot SE	t	*P*	Boot LLCI	Boot ULCI
Constant	2.011	0.142	14.133	< 0.001	1.732	2.290
Age	-0.065	0.046	-1.398	0.162	-0156	0.026
Type of unit	-0.056	0.015	-3.665	< 0.001	-0.087	-0.026
Night shifts per month	0.027	0.030	0.904	0.336	-0.032	0.087
WPB	1.488	0.079	18.777	< 0.001	1.332	1.643
PsyCap	-0.292	0.032	-9.102	< 0.001	-0.355	-0.229
WPB×PsyCap	0.300	0.064	4.702	< 0.001	0.175	0.425
F	6.000					
R^2^	0.372					< 0.001
△R^2^	0.013					< 0.001

Note: Model was adjusted for age, type of unit, night shifts per month

Abbreviations: WPB, workplace bullying; PsyCap, psychological capital; △R^2^, R^2^ change due to interaction term. LLCI, lower-limit confidence interval; ULCI, upper-limit confidence interval

**Table 4:** Conditional effects of nurses’ perceived WPB on emotional exhaustion at different levels of PsyCap (*n* = 1068)

Conditional of PsyCap	Effect	SE	t	*P*	LLCI	ULCI
Mean-1SD	1.200	0.083	14.395	< 0.001	1.037	1.364
Mean	1.488	0.079	18.777	< 0.001	1.332	1.643
Mean + 1SD	1.775	0.114	15.519	< 0.001	1.551	2.000

Abbreviations: WPB, workplace bullying; PsyCap, psychological capital; SD, standard deviation; SE, standard error; LLCI, lower-level confidence interval; ULCI, upper-level confidence interval

**Fig. 2:**
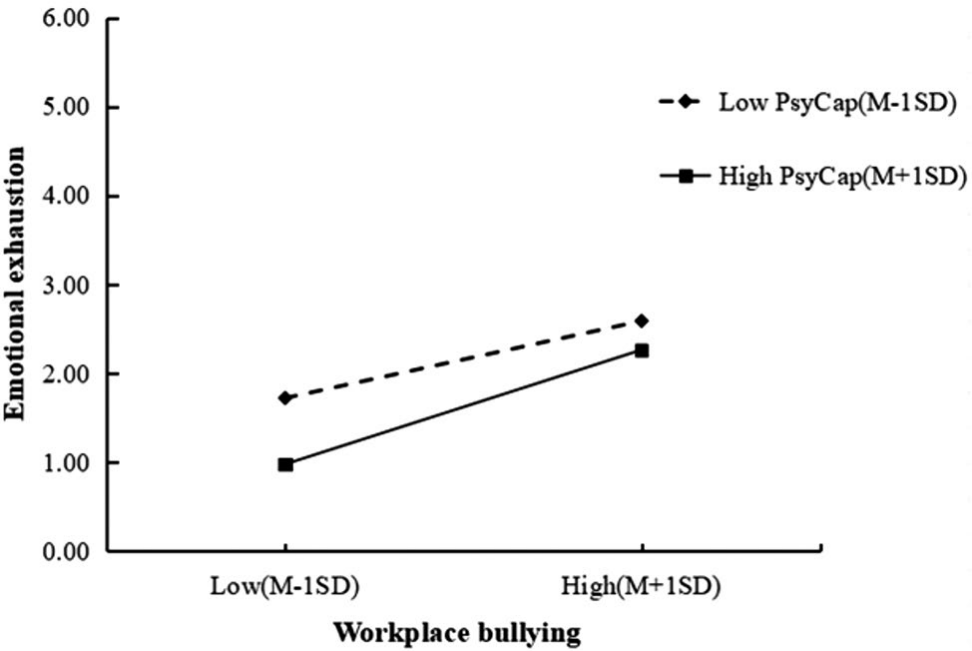
Simple slope test on the moderating effects of PsyCap. Note: PsyCap, psychological capital

To conduct a more in-depth examination of the moderating effects of PsyCap, a simple slope plot was generated (Fig. 2). As shown in Fig. 2, the group with high PsyCap exhibited lower levels of emotional exhaustion compared to the group with low PsyCap under conditions of equivalent WPB exposure. This suggests that PsyCap serves as a buffer against the emotional exhaustion induced by WPB. However, as the perceived WPB increased, the disparity in emotional exhaustion between the high and low PsyCap groups diminished. This trend indicates that the protective efficacy of PsyCap attenuates with WPB increasing.

## Discussion

This study is the first study to explore how PsyCap moderates the relationship between nurses’ perceived WPB and emotional exhaustion, to the best of our knowledge. The findings showed that nurses’ perceived WPB has a positive impact on their emotional exhaustion, and PsyCap moderates this relationship.

This study revealed that 24.0% of nurses reported experiencing WPB within the preceding six months, underscoring the widespread prevalence of bullying in the nursing profession, consistent with prior research [4]. The moderation analysis further corroborated that nurses’ perceived WPB is a risk factor for emotional exhaustion, highlighting the detrimental impact of WPB on nurses’ emotional well-being[16^,^ 17]. Previous studies have already established a significant positive association between WPB and emotional exhaustion, indicating the robustness of this relationship across various cultures and work environments [14–17]. This result confirms that negative work events can trigger negative affective responses, thereby validating the pathway proposed in the AET that work events can elicit affective responses. In light of this, it is imperative for nursing managers to acknowledge the severity and detrimental impact of WPB within the nursing profession. Necessary organizational measures should be taken to mitigate the prevalence of such behaviors, thereby preventing health issues such as emotional exhaustion among nurses. It is particularly advisable to conduct regular assessments of WPB levels within the organization and to analyze high-risk situations that may contribute to WPB due to ineffective management [31, 32]. Additionally, identifying and proactively preventing organizational-level risk factors that may contribute to WPB, cultivating a supportive work environment, and establishing a zero-tolerance policy towards WPB are recommended [31, 33].

The moderation analysis indicated that PsyCap served as a moderating factor in the relationship between WPB and emotional exhaustion, thus supporting our hypothesis. Further examination revealed that PsyCap can mitigate the impact of nurses’ perceived WPB on their emotional exhaustion. These findings validate the moderating role of PsyCap in the relationship between work events and affective responses. Moreover, the results also demonstrate that PsyCap can serve as a job resource to alleviate the adverse effects of high job demands. Consequently, these results not only affirm the robust explanatory power of AET and the JD-R theory but also extend the practical application boundaries of these theoretical frameworks. These results suggest that interventions aimed at enhancing nurses’ PsyCap could be effective strategies to alleviate the negative effects of WPB. For example, implementing micro-intervention training focused on the core components of hope, self-efficacy, resilience, and optimism, as outlined in the Psychological Capital Intervention Model is considered effective in enhancing PsyCap [34]. Additionally, employing positive psychology interventions, such as focusing on strengths, using Ellis Rational-Emotive Therapy, engaging in positive focus and constructive problem-solving activities, and practicing job crafting, has also been regarded as potentially beneficial in this regard [34, 35].

Interestingly, as the perception of WPB increases, the difference in emotional exhaustion between individuals with high and low PsyCap levels decreases. This finding, which has not been previously reported in prior research, indicates that the protective effect of PsyCap decreases as WPB increases in the organizational environment. This phenomenon may occur because perceived WPB is influenced by both individual and organizational factors [36, 37]. When an individual perceives a high level of WPB, there are likely to be more bullying promoting factors within the organization, such as normalization of bullying in nursing teams, organizational tolerance and reward of bullying behaviors [37]. However, PsyCap only serves as an individual protection resource and cannot effectively counteract the negative effects caused by persistent or high levels of WPB within the organization. This finding suggests that in organizations with low levels of WPB, nursing managers can partially rely on nurses’ individual protective resources to mitigate the negative impact on emotional exhaustion. However, in organizations where nurses perceive a higher severity of WPB, nursing managers must address this issue from an organizational and managerial perspective, rather than solely relying on nurses themselves.

This study advances the current understanding of the impact of nurses’ perceived WPB on their emotional exhaustion. Notably, it contributes to the existing body of knowledge by examining how PsyCap can moderate the relationship between WPB and emotional exhaustion among nurses. However, the study has several limitations that should be acknowledged. Firstly, the use of convenience sampling limits the generalizability of the findings and may introduce selection bias. To improve sample representativeness and minimize bias, future research should, where possible, employ random sampling techniques. Secondly, the sample was confined to nurses from tertiary hospitals, which necessitates caution when generalizing the results to nurses in secondary or primary healthcare settings. Replicating the study in these contexts is recommended to ensure broader applicability. Thirdly, the use of the NAQ-R questionnaire, which required participants to recall negative experiences over a six-month period, may have introduced recall bias. Additionally, the cross-sectional design of this study restricts the ability to infer causal relationships. Consequently, future research would benefit from employing longitudinal designs to better assess causality.

## Conclusion

Nurses’ perceived WPB is a risk factor for emotional exhaustion. PsyCap exerts a buffering moderating effect on the relationship between WPB and emotional exhaustion; however, this effect weakens as levels of WPB increase. Thus, developing nurses’ PsyCap helps reduce the negative impact of WPB on bullied nurses. However, in organizational contexts with higher levels of WPB, individuals’ capacity to deal with WPB is limited. Therefore, it is recommended that nursing managers mitigate the detrimental impact of WPB on nurses’ emotional well-being by both strengthening nurses’ individual PsyCap and implementing comprehensive strategies to reduce WPB behaviors.

## Data Availability

Data supporting the findings of this study are available upon request from the corresponding author.

## Abbreviations

AET: Affective event theory
ICU: Intensive care unit
JD-R: Job Demand-Resource
MBI-GS: Maslach Burnout Inventory-General Survey
NAQ-R: Negative Acts Questionnair-Revised
PCQ-R: Psychological Capital Questionnair-Revision
PsyCap: Psychological Capital
WPB: Workplace bullying
